# Repurposing drugs in oncology (ReDO)—selective PDE5 inhibitors as anti-cancer agents

**DOI:** 10.3332/ecancer.2018.824

**Published:** 2018-04-11

**Authors:** Pan Pantziarka, Vidula Sukhatme, Sergio Crispino, Gauthier Bouche, Lydie Meheus, Vikas P Sukhatme

**Affiliations:** 1 Anticancer Fund, Brussels, Strombeek-Bever 1853, Belgium; 2 The George Pantziarka TP53 Trust, London KT1 2JP, UK; 3 GlobalCures Inc., Newton, MA 02459, USA; 4 Emory University School of Medicine, Atlanta, GA 30322, USA

**Keywords:** drug repurposing, PDE5 inhibitors, sildenafil, tadalafil, verdenafil, immunotherapy

## Abstract

Selective phosphodiesterase 5 inhibitors, including sildenafil, tadalafil and vardenafil, are widely-used in the treatment of erectile dysfunction and pulmonary arterial hypertension. They are also well-known as examples of successful drug repurposing in that they were initially developed for angina and only later developed for erectile dysfunction. However, these drugs may also be effective cancer treatments. A range of evidentiary sources are assessed in this paper and the case made that there is pre-clinical and clinical evidence that these drugs may offer clinical benefit in a range of cancers. In particular, evidence is presented that these drugs have potent immunomodulatory activity that warrants clinical study in combination with check-point inhibition.

## Introduction

Phosphodiesterase (PDE) inhibitors are drugs which block the activity of one or more of the 12 PDE isoforms, thereby modulating intracellular levels of cyclic adenosine monophosphate (cAMP) and cyclic guanosine monophosphate (cGMP). PDE5 acts principally on the nitric oxide (NO)/cGMP signalling pathway, clinically important in the treatment of pulmonary hypertension and erectile dysfunction [1, 2]. A range of partially selective PDE5 inhibitors have been developed, including sildenafil, tadalafil, vardenafil, avanafil and udenafil. There are also a number of drugs which have non-selective PDE inhibitory activity, of which some have a degree of PDE5 inhibition, for example, dipyridamole and cilostazol. This review focuses on the anti-cancer properties of the partially selective PDE5 inhibitors, particularly sildenafil, tadalafil and vardenafil.

Sildenafil was the first of the PDE5 inhibitors to be commercially developed, by Pfizer, in the late 1980s. It was originally intended as a treatment for angina pectoris, and entered into the first clinical trials in 1991 [3]. Among the side effects reported in these early studies were penile erections. At that time oral drug treatments did not exist for erectile dysfunction; therefore, as interest in using sildenafil for angina waned, the first trials in erectile dysfunction were initiated in 1993. These early trials were successful and eventually sildenafil, marketed as Viagra, was approved by the Food and Drug Administration (FDA) and European Medicines Agency (EMA) in 1998. Subsequently sildenafil was repurposed as a treatment for pulmonary arterial hypertension [4], and has been investigated as a possible treatment for a number of other conditions, most notably Raynaud’s phenomenon [5].

Tadalafil, trade-marked as Cialis, was also developed as a treatment for erectile dysfunction [6]. In addition the drug is approved in multiple markets for pulmonary arterial hypertension and benign prostatic hyperplasia [7]. Vardenafil and avanafil have EMA and FDA approvals for erectile dysfunction. Udenafil is not FDA-approved, and has orphan drug designation by the EMA for the treatment of functional single ventricle congenital heart disease. It has approval in South Korea and Malaysia for the treatment of erectile dysfunction.

Sildenafil, tadalafil and vardenafil are generic medicines, with multiple manufacturers active in different markets.

## Current usage

### Dosage

The posology of PDE5 inhibitors varies by indication and by drug, as shown in Table 1.

### Toxicity

Common side-effects include dyspepsia, nausea, vomiting, headaches, dizziness, myalgia, back pain and visual disturbances. Less common side-effects can include red eyes, palpitations, tachycardia, hypotension, hypertension and nose bleeds. Rare adverse events have included severe cardiovascular events, sudden hearing loss and retinal vascular occlusion.

PDE5 inhibitors are contra-indicated in patients receiving nitrates or with a previous history of non-arteritic anterior ischemic optic neuropathy. Caution is also advised in patients with cardio-vascular disease, hypotension, recent stroke and left ventricular outflow obstruction.

### Pharmacokinetics

There is significant variation in the pharmacokinetic characteristics of the most widely used PDE5 inhibitors, summarised in Table 2. There are also differences in the degree of PDE5 selectivity. Sildenafil has a high selectivity for PDE5, >1000-fold compared to PDE2, PDE3 and PDE4, but has less so over PDE1 (>80-fold) and PDE6 (>10-fold). Tadalafil is also highly selective for PDE5, >700-fold relative to PDE6, >10,000-fold relative to PDE1–4 and PDE7–10, and >5-fold relative to PDE11. The figures for vardenafil for PDE5 are >15-fold relative to PDE6, >130-fold relative to PDE1, >300-fold relative to PDE11, and >1000-fold relative to PDE2–4 and PDE7–10.

## Pre-clinical evidence in cancer—*in vitro* and *in vivo*

PDE5 inhibition was first shown to induce apoptosis* in vitro* in the SW480 colon tumour cell line using the drug exisulind (an active metabolite of the NSAID COX-inhibitor sulindac) by Thompson *et al*. in 2000 [12]. Exisulind analogues with no cyclo-oxygenase inhibitory activity but increased PDE isoform selectivity showed increased pro-apoptotic activity. PDE5 inhibition using exisulind, E4021 (a specific inhibitor of PDE5), sildenafil, dipyridamole and zaprinast was associated with a reduction in cGMP activity which correlated with apoptosis induction. The same group studied the effect of exisulind in chemically induced (N-butyl-N-(4-hydroxybutyl) nitrosamine) bladder cancer in rats [13]. Results showed that treatment reduced tumour size and multiplicity. Analysis using the human bladder cancer cell line HT1376 showed that the growth inhibitory effect was associated with a reduction in expression of PDE4 and PDE5.

### Chronic lymphocytic leukaemia

In 2003 both sildenafil and vardenafil were shown to cause caspase-dependent apoptosis in patient-derived B-cell chronic lymphocytic leukaemia (B-CLL) cells [14]. The investigation was prompted by the case of a previously untreated man with B-CLL who showed clinically significant improvement following treatment with sildenafil (the patient was treated for erectile dysfunction at a dose of 50 mg/week for a period of 3 months). *In vitro* sildenafil, at a concentration of 50 μg/ml, induced apoptosis in 14 of 14 patient samples. The EC50 (effective concentration of drug that inhibited viability of treated B-CLL cells to 50% of untreated cells), was 4.1 μM for sildenafil and 1.5 μM for vardenafil.

### Prostate

Qian *et al*. [15] investigated the effect of sildenafil in an orthotopic xenograft model of prostate cancer. Male athymic BALB/c nude mice were implanted with PC-3 human prostate cancer cells and treatment with sildenafil, at two different dose levels, or vehicle commencing on day 31 following tumour cell inoculation. Sildenafil was administered by oral gavage at doses of 50 mg/kg or 25 mg/kg, note that the upper dose was calculated to approximate a human sildenafil dose of 100 mg. Results showed no significant effect on primary tumour growth or on metastatic spread when compared to controls. Pernkopf *et al*. [16] later showed that sildenafil, vardenafil and tadalafil had no effect on the proliferation of prostate cancer cell lines *in vitro*, even at high concentrations (1 mg/ml).

A potentiation of therapeutic response to chemotherapy was reported by Das *et al*. [17]. The combination of sildenafil and doxorubicin were assessed *in vitro*. Co-treatment was found to have an additive effect in reducing proliferation and enhancing the rate of apoptosis in PC-3 and DU145 prostate cancer cells, in contrast treatment with sildenafil alone had no effect. *In vivo*, the combination of doxorubicin and sildenafil (at an oral dose of 10 mg/kg) in BALB/c mice bearing human PC-3 prostate cancer xenografts significantly (*P* < 0.05) reduced tumour growth compared to controls. The authors also noted an amelioration of the cardiotoxicity induced by doxorubicin by the addition of sildenafil. Later *in vitro* work by the same group showed that physiologically relevant concentrations of sildenafil, vardenafil and tadalafil enhanced the lethality of a range of chemotherapeutic drugs in a number of gastric cancer cell lines [18].

### Colorectal

Serafini *et al*. [19] used a number of *in vivo* models to demonstrate an immune-mediated anti-tumour effect of sildenafil and tadalafil. BALB/c mice were challenged with CT26WT (colon carcinoma), C26GM (a more aggressive variant of CT26WT) or TS/A (mammary adenocarcinoma) and C57BL/6 with MCA203 (murine fibrosarcoma) cell lines and then treated with the PDE5 inhibitors, starting on the day of inoculation. Treatment reduced tumour growth by 50%–70% compared to controls. Sildenafil treatment commencing on day 7 following inoculation also showed sustained retardation of tumour growth. Experiments in immunodeficient mice showed no difference in tumour growth between mice treated with sildenafil and controls. Additional elucidation of the immune-related mechanisms, (discussed later), was later performed by some of the same authors in a B-cell lymphoma (A20) murine model [20] and by a different group in murine colon cancer and T-lymphoma models [21]. Rigamonti *et al*. [22] also investigated the immune-related effects of sildenafil in two murine models of prostate cancer.

Lin *et al*. [23] showed that PDE5 is over-expressed in human colorectal cancer samples and in azoxymethane/dextran sodium sulphate (AOM/DSS)-induced colon cancers in male BALB/c mice. Intraperitoneal injection of sildenafil (25 mg/kg) for 5 days significantly inhibited PDE5 over-expression, tumour multiplicity and volume in murine AOM/DSS-induced colon cancers compared to untreated controls. Further analysis suggested these positive findings were associated with a reduction in DSS-induced inflammation, specifically a reduction in myeloid-derived suppressor cells (MDSC) infiltration into colonic tissues—a finding also replicated *in vitro*. Islam *et al*. [24] also showed that oral sildenafil (in water, estimated daily dose 5.7 mg/kg) reduced chemically induced polyp formation and colonic inflammation in mice.

Mei *et al*. [25] used a range of human colorectal cancer cell lines, (HT-29, SW480, SW620, HCT116 and SW1116), *in vitro* to assess the effect of sildenafil on proliferation and apoptosis. Results showed IC50 values in the range 190–270 μM. *In vivo* nude mice were implanted with SW480 or HCT116 human cancer cells and treated by oral gavage with sildenafil, either at 50 or 150 mg/kg every 2 days. Tumour volumes were reduced by 40.1% and 57.8% in the SW480 xenografts and by 13.3% and 61.4% in HCT116 xenografts, respectively (*P* < 0.05).

### Brain

Using a rat gliosarcoma (9L) model, Black *et al*. [26] showed that a combination of vardenafil and doxorubicin increased survival compared to untreated controls or either drug alone. Treatment commenced four days after orthotopic implantation of 9L cells in female Fischer rats. Vardenafil was administered orally at a dose of 10 mg/kg, doxorubicin at a dose of 2 mg/kg IV and saline administered to controls. The combination treatment was superior to all three single drug treatments (mean 53 ± 4 days, *P* < 0.05), including doxorubicin alone (mean 42 ± 2 days) which significantly improved survival (*P* < 0.05) compared to control (mean 32 ± 2 days) or vardenafil alone (mean 35 ± 1 days). Subsequently the same group demonstrated improved survival in nude mice bearing cranially-implanted breast and lung cancer tumours, mimicking metastatic spread to the brain, and treated with trastuzumab and vardenafil [27].

Othman *et al*. [28] used primary and recurrent medulloblastoma cell cultures derived from pediatric patients to explore mechanisms of chemoresistance *in vitro*. Cell lines with increased expression of ABCB1 (also known as P-glycoprotein or MDR1) showed relative resistance to *in vitro* treatment with etoposide. However co-treatment with vardenafil (5 and 10 μM) or verapamil increased sensitivity to etoposide.

Roberts *et al*. [29] also determined that co-treatment of a number of medulloblastoma cell lines (D283, DAOY, HOSS 1 and VC312) with sildenafil (2 μM) or tadalafil (2 μM) increased the lethality of standard of care chemotherapy drugs (vincristine, cisplatin and etoposide). Similarly, they showed that sildenafil and tadalafil had additive effects when combined with a non-COX2-inhibitory derivative of celecoxib (OSU-03012) *in vitro* with parental glioma and stem-like glioma cells [30].

### Breast

Di *et al*. [31] reported on the *in vitro* potentiation of doxorubicin cytotoxicity by sildenafil in a panel of breast cancer cell lines, and an *in vivo* reduction in tumour growth rate in a 4T1 breast cancer model (*P* ≤ 0.05), results also confirmed by Greish *et al*. [32].

Roberts *et al*. [33] showed that the combination of sildenafil (0.5 μM) with celecoxib (1 μM) was also cytotoxic *in vitro* in breast, hepatoma, colorectal cancer, glioblastoma and medulloblastoma cell lines. Furthermore, the addition of the multiple sclerosis drug FTY720 (fingolimod), fenretinide or all-trans retinoic acid (ATRA) increased the cytotoxicity of the sildenafil + celecoxib combination. *In vivo*, athymic mice bearing BT474 breast cancer tumours were treated with vehicle, sildenafil (5 mg/kg/day), celecoxib (10 mg/kg/day) or combination for 5 days. The combination showed significantly (*P* < 0.05) lower tumour growth volume compared to single drug treatment. The addition of fingolimod (0.05 mg/kg) slowed tumour growth and increased survival compared to the sildenafil + celecoxib combination (*P* < 0.01).

Sildenafil was also used as an adjuvant in an *in vivo* study of an experimental local tumour ablation modality DaRT (diffusing alpha-emitters radiation therapy) [34]. As with many local ablative therapies, there is some evidence that DaRT can initiate a systemic anti-tumour immune response (abscopal effects) via the release of tumour antigens during local tumour tissue destruction. Confino *et al*. treated BALB/c female mice bearing DA3 undifferentiated breast adenocarcinoma tumours were treated with DaRT, sildenafil, control or the combination (20 mg/kg/day in drinking water for 6 weeks). Combination treatment reduced tumour volume growth compared to single treatments or control (*P* < 0.05). The combination of DaRT, sildenafil and low-dose cyclophosphamide also slowed tumour growth, as did the further addition of CpG.

### Melanoma

Meyer *et al*. [35] employed a *ret* transgenic mouse model of melanoma to investigate the impact of sildenafil on chronic inflammation and the immunosuppressive activity of MDSC. Tumour-bearing mice received sildenafil with drinking water (20 mg/kg/day) for 6 weeks and showed significant (*P* = 0.002) increase in survival compared to untreated controls. This improved survival was associated with inhibition of MDSC immunosuppressive functions and the restoration of T-cell function. The same group also showed that female C57BL/6 mice bearing syngeneic Panc02 pancreatic tumours and treated with sildenafil in drinking water (20 mg/kg/day) had increased survival compared to untreated mice (*P* < 0.01), however male mice showed a trend towards decreased survival [36].

### Multiple myeloma

Kumazoe *et al*. [37] showed that a range of PDE5 inhibitors enhanced the apoptotic effects of the green tea polyphenol (–)-epigallocatechin-3-O-gallate (EGCG) in a panel of multiple myeloma (MM) cell lines. Sildenafil (10 μM) and vardenafil (5 μM) significantly reduced the viability of U266, ARH77 and RPMI8266 cell lines pre-treated with EGCG (5 μM) compared to EGCG alone (*P* < 0.001). Data from primary MM cells, (*n* = 10), showed that EGCG and vardenafil alone had little impact on viability, but that the combination reduced viability to a similar degree to the MM cell lines. Similar *in vitro* results were shown for MKN45 (gastric cancer), PANC-1 (pancreatic cancer), and PC3 (prostate cancer) cell lines. *In vivo*, female BALB/c mice injected with murine MM cells (MPC-11) were given treated with intraperitoneal injections of EGCG (15 mg/kg) and/or vardenafil (5 mg/kg) every 2 days data. Mice treated with the combination showed significantly reduced tumour volume (*P* = 0.019) and improved survival (*P* < 0.001). Subsequently the same group showed similar *in vitro* results, in primary patient samples and established cell lines, with the combination of EGCG and vardenafil in acute myeloid leukaemia and chronic lymphocytic leukaemia (CLL) [38, 39].

### Lung

Sildenafil, vardenafil and dipyridamole were investigated by Li and Shu [40] as potential enhancers of response to cytotoxic chemotherapy drugs in the H1915 lung cancer cell line and in a murine model. *In vitro* results showed that all three drugs tested, (at concentrations: 20 μM vardenafil, 100 μM sildenafil and 20 μM dipyridamole), increased the uptake of doxorubicin and carboplatin. Vardenafil at 20 μM also reduced the viability of H1915 cells when treated with a range of doses of doxorubicin and carboplatin. For the *in vivo* experiments, using athymic nude mice, only vardenafil was used, at an oral dose of 10 mg/kg for 5 days per week. As with the *in vitro* results, vardenafil increased the tumour accumulation of dextran, trastuzumab and doxorubicin, although only the first two results were significant (*P* < 0.05). Furthermore, combination treatment of vardenafil and trastuzumab significantly slowed tumour growth compared to either drug alone or to untreated controls.

Booth *et al*. [41] showed the synthetic lethality of the combination of sildenafil, vardenafil or tadalafil with pemetrexed in non-small cell lung cancer (NSCLC) cell lines and *in vivo* of the combination of sildenafil (5 mg/kg) and pemetrexed. The work has also been extended to show that the combination of sildenafil, pemetrexed and sorafenib enhances the *in vivo* anti-tumour effects of the pemetrexed + sorafenib combination in a H460 NSCLC model [42], the combination of celecoxib, sildenafil and sorafenib in a panel of ovarian cancer cells lines [43] and pemetrexed, sildenafil and sodium valproate in NSCLC and ovarian cell lines [44]. Domvri *et al*. [45] also showed *in vitro* that 100 μM sildenafil increased the response to carboplatin in the H1048 SCLC cell line and the A549 NSCLC cell line.

### Lymphoma

Wang *et al*. [46] investigated whether tadalafil modulated rituximab activity in a murine model of brain lymphoma. Raji human lymphoma cells were implanted in the cranium of athymic mice and allowed to form and then mice were treated with saline, tadalafil at 1.5 mg/kg (oral gavage at twice per week), rituximab 30 mg/kg, (via tail vein injection twice per week) or combination of tadalafil and rituximab. Survival analysis showed that tadalafil alone was similar to untreated control, rituximab alone was significantly (*P* = 0.01) superior to tadalafil alone and that the combination of tadalafil and rituximab was significantly (*P* = 0.03) better than rituximab alone (rituximab group, 25.75 ± 1.892 days; and tadalafil + rituximab group, 29 ± 1.414 days).

### Liver

Sildenafil was also investigated pre-clinically by Tavallai *et al*. [47] as a potential synergist with sorafenib and regorafenib in liver and colorectal cancer. Sildenafil (2 μM) in combination with sorafenib and regorafenib increased cell death in hepatoma cell lines (HEP2G, HEP3B and HuH7) *in vitro*. *In vivo* athymic mice bearing HuH7 (hepatoma), HCT116 (colorectal) and BT474 (breast) tumours were treated with vehicle, sildenafil (5 mg/kg), regorafenib (25 mg/kg) or the combination. Combination treatment significantly reduced tumour growth (*P* < 0.05) compared to regorafenib alone. The combination of sildenafil and sorafenib reduced the increase in tumour volume compared to sorafenib alone in mice bearing HuH7 tumours. The addition of fingolimod to the combination of sildenafil and regorafenib in HT29 colon cancer models increased tumour regrowth after the cessation of drug treatment compared to the sildenafil and regorafenib combination.

### Head and neck

In addition to showing that tadalafil and sildenafil reduced cell viability in a panel of head and neck squamous cell carcinoma (HNSCC) lines (UM1, UM6, UM47 and CAL27), Tuttle *et al*. [48] also explored the effect in an *in vivo* model. Athymic (nu/nu) mice inoculated with CAL27 cells were treated with tadalafil (via osmotic pumps delivering 1 mg/kg/day) or vehicle. Tumour weight and volume were significantly reduced compared to controls (*P* < 0.05).

Sponziello *et al*. [49] showed that PDE5A was overexpressed in a series of human papillary thyroid carcinomas compared to normal tissues. Expression was higher in samples with BRAF V600E mutation compared to wild-type BRAF. Furthermore it was shown that sildenafil and tadalafil reduced proliferation and migration in BCPAP, TPC-1 and 8505C thyroid cancer cell lines. The anti-proliferative effects could be enhanced through the use novel nano-formulations of the two drugs [50].

### Rhabdomyosarcoma

Zenitani *et al*. [51] investigated the combination of sildenafil and C-type natriuretic peptide (CNP), an endogenous peptide secreted by vascular endothelial cells, pre-clinically in pediatric rhabdomyosarcoma (RMS) [51]. While CNP showed anti-proliferative effects on two RMS cell lines, sildenafil alone showed an anti-proliferative effect in the cell RMS-YM-GC-B cell line but not the RD-GC-B cell line (all *P* < 0.05). However, sildenafil enhanced the anti-proliferative effects of CNP in both cell lines compared to either single agent treatment (*P* < 0.05). *In vivo* the combination of CNP and sildenafil (20 mg/kg, intraperitoneally every second day) attenuated tumour weight and volume compared to vehicle control (*P* < 0.05).

### Ehrlich ascites carcinoma

El-Naa *et al*. [52] used an *in vivo* ehrlich ascites carcinoma (EAC) model to demonstrate synergy between cisplatin and sildenafil. Sildenafil was administered in drinking water at a dose of 5 mg/kg/day for 15 days following inoculation with EAC cells. Single agent treatment with sildenafil and cisplatin showed a significant reduction in tumour volume growth compared to untreated controls (*P* < 0.05), and the combination of sildenafil and cisplatin showed significant reduction compared to both single agent treatments (*P* < 0.05).

The pre-clinical evidence, both *in vitro* and *in vivo*, is summarised in Table 3.

## Human evidence

The first report of anticancer activity of a PDE5 inhibitor in humans was published in 2004 [53]. Treon *et al*. published a report of five cases in which patients suffering from Waldenstrom’s macroglobulinemia, an uncommon and incurable B-cell malignancy, responded to treatment with sildenafil. The initial case was an 80-year old man who showed a complete response upon commencing sildenafil treatment for erectile dysfunction. On noting this unexpected outcome, four further cases were discovered in the same clinic, all four cases showing reductions in serum immunoglobulin M (IgM) levels—although none showed the complete response of the first patient. Additional *ex vivo* analysis showed evidence that sildenafil, at a concentration of 0.01 μg/mL, caused apoptosis in lymphoplasmacytic cells from the five patients.

Based on these incidental findings a small Phase II open-label clinical trial (NCT00165295) was initiated [54]. Patients, (*n* = 30, 18 of whom were treatment naive), with slowly progressive Waldenstrom’s macroglobulinemia and ineligible for active therapy were treated with 100 mg/day of sildenafil, with a starting dose of 25 mg/day and escalating to the target dose over a 4 week period. At a median of 3 months, serum IgM levels declined in 19/30 (63%) patients by around 18% and 5/30 patients (17%) demonstrated at least a minor response (≥25% IgM decrease).

An incidental finding that sildenafil has an effect on severe lymphatic malformations was also reported by Swetman *et al*. [55]. A 10-week old child suffering from pulmonary hypertension as a consequence of severe lymphatic malformations in the chest was treated with sildenafil to address the symptoms. After commencing treatment the malformation diminished and was subsequently confirmed by MRI. Based on this case two more children were treated and also showed reduction in the extent of the lesions and improvements in functioning. Gandhi *et al* also reported on two cases of pediatric orbital lymphangioma which responded to sildenafil treatment [56]. Although these are benign lesions, it is instructive to compare these results to the incidental finding that propranolol was effective in the treatment of infantile hemangioma and other highly vascularised benign tumours, subsequently leading to the investigation of propranolol in angiosarcoma [57].

Huilgol and Jain [58] reported on three patients with penile squamous cell carcinoma who were treated with sildenafil as a radiosensitiser. The authors hypothesised that the increased blood flow due to sildenafil when used for erectile dysfunction would be beneficial in reducing hypoxia and hence improving response to radiotherapy. The men were treated with sildenafil at 50 mg/day 5 days a week, administered 30 minutes prior to radiotherapy and all three completed planned treatment. One patient suffered local recurrence at 10 months and succumbed to disease at 23 months. The other two patients were disease free at 53 and 48 months respectively.

Noonan *et al*. [59] reported on a patient with end-stage MM treated with tadalafil in combination with lenalidomide, clarithromycin and dexamethasone. The 50-year old man had been treated with multiple regimens after recurrence following near complete remission with VAD (vincristine, doxorubicin and dexamethasone) induction therapy and autologous stem cell transplant. He had been treated with lenalidomide, dexamethasone and clarithromycin but had to stop due to intolerance. However, with the addition of tadalafil the patient was able to tolerate lenalidomide and dexamethasone, and with the addition of the antibiotic clarithromycin (which has evidence of anti-myeloma activity [60]), the patient achieved a very good partial response (near 90% reduction in disease burden). Analysis showed a reduction in MDSC numbers and a restoration of T cell function.

Beneficial impacts of tadalafil were also shown in a small (*n* = 35) randomised clinical trial (NCT00843635) in HNSCC [61]. Patients were randomised to tadalafil at 10 mg/day, 20 mg/day or placebo for at least 20 days pre-operatively (drug was stopped 36 hours prior to surgery). Tadalafil significantly reduced MDSC and T reg numbers in the blood of patients, with optimal immunomodulatory response between a dose of 145 and 225 μg/kg. It was noted that the immunomodulatory effect was blunted at higher concentrations, possibly due to off-target inhibition of PDE11 by tadalafil. A separate randomised controlled trial in HNSCC patients (NCT00894413) at the same institution showed that tadalafil, at a dose of 20 mg/day, significantly decreased peripheral MDSC numbers and increased general immunity as measured by delayed type hypersensitivity response (*P* < 0.002) [62].

Building on prior work that showed that chronic inflammation, MDSCs and T reg cell numbers were associated with a worse prognosis in advanced melanoma [63], and *in vivo* studies in a transgenic mouse model [35], a small pilot trial (*n* = 12) of tadalafil was carried out in palliative care patients with metastatic melanoma [64]. The majority of patients (11/12) in the TaMe trial were heavily pre-treated with a range of interventions, including checkpoint inhibitors and chemotherapy; one patient had not been pre-treated due to a contra-indication to ipilimumab. A dose de-escalation design was used and four dose levels tested: 5, 10, 20 and 40 mg/day. Tumour and peripheral blood samples were taken before and 4 weeks after the start of treatment to assess immunological response, with change in CD8+ tumour infiltrating lymphocytes (TILs) as primary end point. Stable disease was achieved in 3/12 patients (25%), with median progression-free survival (PFS) of 4.6 months (range 0.7–7.1) and overall survival (OS) 8.5 months (range 2.7–23.7). Patients with stable disease displayed significantly higher pre-treatment CD8+ TILs in the centre of metastases and after therapy showed increased expression of ζ-chain in CD8+ and CD4+ TILs and CD8+ T cells in the peripheral blood. Metastases of stable patients were also characterised by a significant reduction of FOXP3+ T reg cells post-treatment as compared with baseline. There was no relationship between stable disease and tadalafil dose.

In addition to these examples of possible anticancer effects of PDE5 inhibitors, there have also been concerns that chronic use of sildenafil and related drugs may be associated with an increased risk of cancer incidence. Based on data that showed that low PDE5A expression increased the invasiveness of melanoma cells [65], Li *et al*. [66] conducted a large prospective cohort study in US men to assess the association between sildenafil use for erectile dysfunction and incidence of melanoma and other skin cancers. The authors reported an elevated risk of melanoma hazard ratio (HR), 1.92; 95% confidence interval (CI) 1.14—3.22] but no increased risk of squamous cell carcinoma or basal cell carcinoma. A subsequent Swedish analysis by Loeb *et al*. [67] also reported an association, but raised questions as to causality. A UK analysis also found a small association, (HR = 1.14, 95% CI 1.01–1.29, *P* = 0.04), but the data suggested that men with higher sun exposure were more likely to become PDE5 inhibitor users [68]. Other, more recent, datasets have also cast doubt on a causal effect of sildenafil and other PDE5 inhibitors on melanoma risk [69, 70].

A meta-analysis by Tang *et al*. [71] concluded that PDE5 inhibitor use was associated with a slightly elevated risk of melanoma (OR, 1.12; 95% CI 1.03–1.21) and basal cell carcinoma (OR, 1.14; 95% CI 1.09–1.19) but not squamous cell carcinoma, however they concluded that causality remained elusive. A meta-analysis by Loeb *et al*. [72] also concluded that there was an elevated risk, but that the data showed a lack of dose response suggesting the relationship was not causal.

Concerns were also raised that long-term PDE5 inhibitor use might increase the risk of biochemical relapse after radical prostatectomy for localised prostate cancer [73]. Michl *et al*. reported that use of a PDE5 inhibitor after radical prostatectomy was associated with an increased risk of biochemical recurrence (HR 1.38, 95% CI 1.11–1.70, *P* = 0.0035). However, a more recent study has reported no such association [74] and a retrospective study indicated a trend towards PDE5-inhibitor mediated protective effect against primary prostate cancer [75].

## Clinical trials

As of 24 August 2017, a number of clinical trials are investigating the anti-cancer uses of sildenafil, tadalafil and vardenafil, as shown in Table 4. Note that trials which are assessing non-cancer-related outcomes, for example trials such as NCT01375699 which is assessing the cardio-protective effect of sildenafil on doxorubicin-treated cancer patients are not included. Only trials which are currently open (recruiting or soon to commence recruitment) or on-going are included.

## Mechanisms of action

The role of PDE5 in different tissues and diseases is reviewed by Kouvelas *et al*. [76], here we will focus on the different mechanisms of action which have been explored in relation to the anti-cancer effects of the PDE5 inhibitors discussed in this review.

### Immunological

Serafini *et al*. [19] first outlined the immunomodulatory effects of PDE5 inhibitors in relation to cancer in 2006. In particular they showed that in a number of murine models tadalafil and sildenafil significantly delayed tumour growth in immune competent mice but not immunocompromised mice. Additionally they determined *in vitro* that these drugs had no impact on apoptosis in the relevant cell lines. Further experiments demonstrated that sildenafil enhanced the tumour-specific T cell response and increased intra-tumour CD8+ T cell infiltration and activation. *In vivo* PDE5 inhibition reduced ARG1 and NOS2 and down-regulated IL-4Rα in tumour-associated MDSCs in BALB/c and C57BL/6 tumour-bearing mice. It was also shown that expansion of CD4+ and CD8+ T cells obtained from MM or head and neck cancer patients was significantly lower than that observed using healthy donors. However, in the presence of sildenafil, PBMCs from these patients expanded similarly to sildenafil treated PBMCs from healthy donors. Subsequently the same group explored the role of MDSCs in T cell anergy in the A20B-cell lymphoma model [20]. It was shown that MDSCs induce the proliferation of regulatory T cell (Tregs) and the establishment of T cell tolerance. Sildenafil treatment reduced the number of tumour-specific Tregs, and reverted tumour-induced T-cell anergy.

Capuano *et al*. [21] confirmed that sildenafil inhibited the development of an immunosuppressive phenotype in two murine models of colorectal cancer by downregulation of MDSC cell populations. Analysis showed the effects were mediated by inhibition of iNOS and arginine metabolism. Similar results were shown in C57BL/6 mice challenged with TRAMP-C1 cells where oral sildenafil retarded tumour growth compared to vehicle-treated controls [22]. However, while sildenafil reduced MDSCs numbers and affected arginine metabolism in spontaneous tumours in TRAMP mice but did not significantly retard tumour growth.

As discussed previously, Meyer *et al*. [35] showed that the inflammatory tumour microenvironment was associated with MDSC-mediated immunosuppression in melanoma-bearing mice. They showed that MDSC reduced both T cell proliferation and ζ-chain T cell receptor expression, thereby creating an immunosuppressive tumour microenvironment [77]. Treatment with sildenafil reduced MDSC numbers infiltrating primary tumours and metastatic lesions, increased CD8+ T cells and induced a partial recovery of ζ-chain expression. Sildenafil treatment was also associated with improved survival in tumour-bearing mice. Lin *et al*. [23] also showed that sildenafil inhibited the development of colitis-associated colonic tumours in BALB/c mice by inhibiting MDSC infiltration into colon tissues.

Booth *et al*. [44] have also shown that the combination of sildenafil and pemetrexed enhanced immunogenic cell death in a syngeneic mouse Lewis Lung Carcinoma model. *In vitro* the combination treatment increased PD-L1 and ODC expression in tumour cells and enhanced the expression of MHCA and HMGB1. *In vivo* combination treatment with pemetrexed and sildenafil enhanced the anti-tumour efficacy of checkpoint inhibitory antibodies directed against programmed cell death protein 1 (PD-1) or cytotoxic T-lymphocyte-associated protein 4 (CTLA4).

A recent review has summarised the diverse effects of sildenafil in both the innate and adaptive immune systems [78].

### Treatment sensitisation

Multidrug resistance protein 5 (MRP5/ABCC5) was shown to confer resistance to the commonly used cancer drug 5-FU in human embryonic kidney cells *in vitro*, increasing the EC50 value by a factor of ten in cells transfected with MRP5 compared to non-transfected cells [79]. It was further shown that a number of agents, including sildenafil, could reduce the ATP-dependent transport of active 5-FU metabolites.

Shi *et al*. [80, 81] showed that sildenafil increased the *in vitro* sensitivity of ABCB1-overexpressing drug-resistant cells to colchicine, vinblastine and paclitaxel but not cisplatin. Sildenafil was also shown to increase the accumulation of paclitaxel. Furthermore, in ABCG2-overexpressing cells, sildenafil inhibited resistance to ABCG2 substrate anticancer drugs, for example, mitoxantrone. It was noted also that sildenafil exhibited greater affinity to ABCB1 than to ABCG2. Some of the same authors also showed that vardenafil, and to a lesser extent tadalafil, also increased the sensitivity of ABCB1 over-expressing cells but did not alter the drug sensitivity in ABCC1 and ABCG2 overexpressing cells [82]. MRP7/ABCC10 is another of the MRP family is associated with resistance to a number of chemotherapeutic agents, including vinca alkaloids and taxanes, which has been shown to be amenable to PDE5 inhibitor-mediated sensitisation [83].

A study into the potential clinical use of sildenafil to address treatment sensitivity was published by Lin *et al*. [84] following the range of *in vitro* results described above. In a series of experiments using wild-type and ABC-transporter knock-out they showed that sildenafil (50 mg/kg) did not increase brain accumulation of topotecan or docetaxel. A second set of experiments using CT26 tumours in syngeneic BALB/c mice showed that sildenafil (10 and 50 mg/kg) increased tumour accumulation of doxorubicin, but also increased accumulation in plasma and heart tissue at the higher dose. However, this did not translate into a reduction in tumour volume compared to doxorubicin alone.

Das *et al*. [17, 85] also showed a potentiation of chemotherapeutic response via the increased generation of reactive oxygen species (ROS) using the combination of sildenafil and doxorubicin in mouse models of prostate cancer. In addition to showing that the drug combination additively increased apoptosis of PC-3 and DU145 prostate cancer cells, they also showed that in nude mice carrying PC-3 flank tumours the combination reduced tumour volume. Mechanistically the addition of sildenafil to doxorubicin increased ROS production in both PC-3 and DU145 cell lines though not in PrEC normal cells. A key role for CD95 was also identified [85]. Subsequent studies by the same authors assessed the combination of PDE5 inhibitors with standard chemotherapeutic drugs in a range of bladder and pancreatic cancer cells [18]. Bladder cancer cells (HT-1376, J82 and T24) showed increased levels of cell death when treated with the combination of sildenafil and mitomycin C, doxorubicin, cisplatin or gemcitabine compared to treatment without the addition of sildenafil. Similarly pancreatic cancer cells (PANC-1, Mia, Paca2 and AsPC-1) were more sensitive to combination of sildenafil and doxorubicin, gemcitabine or paclitaxel. The effect was mediated by the induction of ROS and an increase in DNA damage.

Sildenafil and vardenafil were also shown to enhance tumour permeability via effects on the blood brain barrier [26]. Specifically looking to address the *blood–brain tumour barrier*, which includes the microvessel supplying brain tumours, Black *et al*. showed that oral sildenafil (50 mg/kg) and vardenafil (10 mg/kg) increased tumour permeability of radioactively labelled sucrose. Furthermore, 9L gliosarcoma-bearing rats treated with the combination of vardenafil and doxorubicin had significantly (*P* < 0.05) longer survival than animals treated with either agent alone or vehicle control. Wang *et al*. [46] showed that tadalafil improved microvascular permeability leading to an improved survival in a murine brain lymphoma model treated with rituximab.

### Hypoxia

Tumour hypoxia is associated with resistance to treatment in solid tumours, increased genomic instability and the development of a metastatic phenotype in multiple cancer types [86]. It has been shown that hypoxia-induced resistance to doxorubicin could be reversed with the use of NO mimetics such as nitroglycerin [87]. Bell *et al*. [88] showed that atrial natriuretic peptide, one of a class of polypeptides that cause diuresis/natriuresis and vasodilation, inhibited the hypoxia-induced resistance to doxorubicin in prostate cancer cell lines (DU-145 and PC-3). The same authors subsequently explored the potential of PDE5 inhibitors to address the same issue [89]. They showed that in DU-145 and PC-3 prostate cancer cell lines PDE5 and PDE11 were responsible for cGMP-specific PDE activity and that chemical inhibition of PDE5 with zaprinast was associated with reduced hypoxia-induced resistance to doxorubicin *in vitro* and reduced tumour growth *in vivo*.

Ikeda *et al*. [90] showed that hypoxia induced resistance to the novel anticancer agent Poly-SNO-HAS (poly-S-nitrosated human serum albumin) in murine colon 26 adenocarcinoma (C26) cells *in vitro* and *in vivo*. However, the combination of vardenafil and Poly-SNO-HAS showed increased tumour growth reduction compared to either drug alone or control under hypoxic conditions.

Ammirante *et al*. [91] showed that hypoxic conditions in the androgen-deprived prostate tumour microenvironment activates cancer-associated fibroblasts, induces TGF-β expression and stimulates CXCL13 production—a process associated with a more aggressive tumour phenotype. Treatment with sildenafil significantly delayed the emergence of castration-resistance in castrated Myc-CaP tumour-bearing mice by inhibiting the hypoxia-induced activation of fibroblasts. This result is notable given that tadalafil is already approved for the treatment of benign prostatic hyperplasia.

### Other

In addition to immunological and chemo-sensitivity mechanisms, there is some evidence for a range of other effects that may be relevant in the context of cancer treatment—many of these effects are driven by the inhibition of PDE5 activity in cancer cells. Zhu and Strada [92] summarised the increased activity of PDE isoforms in a range of colon, breast, prostate, lung and other cancer cell lines and showed that PDE5 was the most commonly over-expressed isoform. They also showed that PDE5 inhibition with siRNA or exisulind in colon cancer cell lines was associated with mitotic arrest and the induction of apoptosis.

Karami-Tehrani *et al*. [93] compared PDE5 and PDE9 expression in normal breast tissue, benign and malignant breast tumour samples. The relative expression of both PDE isoforms in malignant tumours was significantly higher than those of respective normal breast tissues and benign tumours, with evidence of an association between overexpression and tumour grade, stage, and lymph node involvement. Catalano *et al*. [94] assessed PDE5 expression in breast cancer cell lines and found higher expression in HER2-overexpressing (SKBR3) and basal-like (BT-20/MDA-MB-468/MDA-MB-435) lines, suggesting a correlation between PDE5 expression and more aggressive disease. PDE5 over-expression *in vitro* was associated with an increase in cell motility and migration—which could be reversed by sildenafil. Retrospective analysis showed that high levels of PDE5 expression were associated with shorter overall survival in breast cancer patients (*P* = 0.014, HR = 1.2) [94].

The combination of CNP and sildenafil was shown to reduce growth rate in xenograft RMS tumours via sildenafil-mediated inhibition of degradation of CNP-induced accumulation of cGMP [51]. *In vitro* analysis suggested that the combination exerted anti-proliferative effects on RMS cells by inhibiting the Raf/MEK/ERK pathway.

Liu *et al*. [95] investigated the role of the Hippo pathway in PC3-derived prostate cancer stem cells (PCSC). The showed that PCSCs showed expressed 2.8-fold more PDE5 than non-stem prostate cancer cells and that PDE5/cGMP/PKG signalling was a key component of PCSC-related Hippo/TAZ pathway. *In vitro* inhibition of PDE5 signalling using vardenafil attenuated ‘stemness’ and dose dependently suppressed colony formation in both PC3 cells and PCSC. *In vivo* vardenafil reduced PSCS stemness and increased apoptosis. Similarly, Booth *et al*. [96] have shown that sildenafil synergised with celecoxib or the celecoxib derivative OSU-03012 to increase cell death of a range of glioblastoma stem-like cells.

Finally, a recent report by Baravalle *et al*. [97] used molecular and cell level analytics to show that PDE5 inhibition also acts to inhibit aromatase, an important target in breast cancer. Sildenafil was found to act as a partial inhibitor of human aromatase, with a maximal inhibition of around 35%.

## Our take

The evidence from *in vitro*, *in vivo* and human studies, outlined above and summarised in Table 5, suggests that the licensed PDE5 inhibitors sildenafil, vardenafil and tadalafil have a number of distinct anti-cancer effects which may be of therapeutic value in different clinical settings. While a number of distinct mechanisms of action have been elucidated, the immunological and treatment sensitisation aspects of these drugs are especially intriguing. We note the encouraging number and range of on-going clinical trials exploring these aspects of the anti-cancer activity of PDE5 inhibition. In particular these drugs show potential as adjuncts to existing anticancer therapies via two distinct mechanisms.

Firstly by synergising or potentiating the therapeutic effects of other agents, including a wide range of chemotherapeutics and other drugs. Reversing or reducing resistance to existing treatments—including radiotherapy, chemotherapy and endocrine therapy—is an important means of improving patient outcomes. This is particularly the case for brain tumours, both for primary tumours and metastases, where the blood-brain-barrier means there are few therapeutic agents that have proven efficacy. The use of drugs to increase permeability is an active area of research and one in which PDE5 inhibitors show great potential.

Secondly, by addressing the underlying mechanisms of immunosuppression through modulation of MDSCs and/or Treg cell populations. MDSCs play a key role in suppression of anti-cancer immune responses and are a high-value therapeutic target in oncology [98, 99]. Indeed, MDSCs are a key target in addressing resistance to immune checkpoint inhibitors [99]. PDE5 inhibitors are not the only repurposing candidates which have some evidence of activity related to MDSCs and Treg cells, others include histamine type-2 receptor antagonists such as cimetidine [100] and ranitidine [101], metronomic cyclophosphamide [102] or NSAIDs such as aspirin or celecoxib [103]. The potential synergism of these low-cost and low-toxicity treatments is intriguing and warrants both pre-clinical and clinical investigation. The inclusion of PDE5 inhibitors with checkpoint inhibition, as with the TONIC-trial of metronomic cyclophosphamide and nivolumab [104], and in the NCT03238365 trial of tadalafil and nivolumab is also an exciting prospect.

The treatment sensitisation and immunological effects are not mutually exclusive. PDE5 inhibitors therefore offer the attractive prospect of increasing immunogenic cell death while at the same time enhancing the immune response. The combination with immune checkpoint inhibitors is therefore particularly worthy of investigation.

Perioperative therapies are interventions designed to improve the outcomes from cancer surgery by reducing post-surgical recurrence rates [105, 106]. Interventions that have been explored include pre-operative ketorolac [107] or aspirin [108], depot progesterone [109], cimetidine [110] and immunonutrition with l-arginine [111]. There is some evidence, both pre-clinical and clinical, that perioperative vaccination with influenza vaccine may reduce post-surgical metastatic spread by enhancing natural killer cell number and cytotoxicity [112]. The PERIOP-04 (NCT02998736) trial is assessing the combination of pre-operative tadalafil and influenza vaccine in patients undergoing surgical resection for abdominal malignancies. Given the range of mechanisms which may be implicated in post-surgical locoregional and distant recurrence there is reason to believe that multiple interventions may be required to fully reduce the risks. Therefore it is proposed that multi-arm, multi-stage platform trials should be explored as a mechanism to explore the relative efficacy of different perioperative therapies and their combinations.

### Next steps

As outlined previously, there are a number of active Phase I and Phase II clinical trials on-going at present. These trials are largely based on to the strong level of clinical evidence in a number of specific indications and it is to be hoped that positive reports from these trials will be forthcoming in the future. The data are strongest for clinical trials of PDE5 inhibitors, in combination with other agents, in the following cancer types:

- HNSCC
- Glioblastoma
- Pancreatic cancer
- Medulloblastoma
- Waldenstrom’s macroglobulinemia
- Melanoma

The perioperative use of PDE5 inhibitors in combination with other perioperative therapies is also of interest in the following cancers:

- Colorectal cancer
- Breast cancer
- HNSCC

The diagnostic and predictive significance of PDE5 expression in oncology is also an area that warrants additional attention. This is particularly the case in a precision oncology context where patient selection on the basis of PDE5 expression may be required.

## Conclusions

A broad range of data, pre-clinical and clinical, has been summarised and presented to make the case that the commercially available and widely used PDE5 inhibitors sildenafil, vardenafil and tadalafil are very strong candidates for repurposing as anticancer agents. These low-cost, low-toxicity drugs show potential to be included with current and emerging standard of care treatments in oncology. The combination with immune checkpoint inhibitors or possible use as perioperative therapies are particularly compelling strategies with the potential to positively improve survival outcomes in a relatively short time-frame.

## Author contributions

Primary author: Pan Pantziarka. Contributing authors: Vidula Sukhatme, Sergio Crispino, Gauthier Bouche, Lydie Meheus and Vikas P. Sukhatme. All authors read and approved the final manuscript.

## Competing interests

The authors declare that they have no competing interests. All of the authors are associated with not for profit organisations that aim to repurpose drugs for oncology treatments. VPS is also a scientific advisory board member of Berg Health and Mitra Biotech.

## Figures and Tables

**Table 1.** Posology of most commonly used PDE5 inhibitors.

Indication	Sildenafil	Tadalafil	Vardenafil
Erectile dysfunction	25–100 mg, single dose as needed	10–20 mg, single dose as needed. 5 mg/day for regular use	10–20 mg, single dose as needed
Pulmonary arterial hypertension	5–20 mg, three times daily	40 mg, once daily	
Benign prostatic hyperplasia		5 mg, once daily	

**Table 2.** Pharmacokinetics of sildenafil, tadalafil and vardenafil.

	Sildenafil (100 mg oral dose) [8–10]	Tadalafil (20 mg oral dose) [11]	Vardenafil (20 mg oral dose) [11]
PDE5 IC50	3.9 nM	0.94 nM	0.1–0.7 nM
Oral bioavailability	41%	36%	15%
Peak plasma concentration	1.18 μM	0.80 μM	0.04 μM
Time to peak	1.5 hours	2.0 hours	0.7 hours
Plasma half life	3–5 hours	17.5 hours	4–5 hours

**Table 3.** Summary of *in vitro* and *in vivo* evidence.

Cancer Type	Drug	Comment
**Breast**	S	*In vitro* increase in doxorubicin cytotoxicity, *in vivo* reduction in tumour growth rate [31, 32].
	S	*In vitro* combination with celecoxib cytotoxic, *in vivo* combination with celecoxib increased survival [33].
	S	*In vivo* combination with local ablation reduced volume of tumour growth [34].
**B-CLL**	S, V	Both drugs induced apoptosis in 14 of 14 patient samples [14].
**Colorectal**	S, T	*In vivo*—both drugs reduced tumour growth via immune-mediated effects [19, 21].
	S	*In vitro*, *in vivo*—reduction in colon tumour growth [23–25]
	S	*In vitro*—combination with celecoxib cytotoxic [33].
**EAC**	S	*In vivo*, reduced tumour growth alone and in combination with cisplatin [52].
**Gastric**	S, D, V	*In vitro*—synthetic lethality in combination with standard chemotherapy drugs in bladder cancer cell lines [18].
**Glioma**	S, V	*In vivo*—drugs increase permeability of blood-brain-tumour barrier, increased survival in combination with doxorubicin [26].
	V	*In vitro*, *in vivo*—brain metastases models (cranial breast and lung cancer implants) show increased survival in herceptin treated mice [27].
**HNSCC**	S, T	*In vitro* both drugs reduce viability in HNSCC cell lines. *In vivo* T reduces tumour growth in xenograft model [48].
**Liver (hepatocellular carcinoma)**	S	*In vitro*, *in vivo*—combination with sorafenib or regorafenib show synergism in reducing tumour growth [47].
**Lung**	S, V	*In vitro* both drugs increased update of doxorubicin and carboplatin in lung cancer cells. *In vivo* V increased update of trastuzumab and decreased tumour growth [40].
	S	*In vitro*, *in vivo* combination with pemetrexed and sorafenib enhances cytotoxicity in lung cancer cell lines and athymic mouse model [42].
	S	*In vitro* both SCLC and NSCLC cell lines showed increased apoptosis when treated with sildenafil and carboplatin [45].
**Lymphoma**	S	*In vitro*, *in vivo*. Reduction of MDSC and T reg cells—preventing T cell energy [20] and reduced tumour growth [21].
	T	*In vivo*—combination with rituximab improved survival in a murine model of brain lymphoma [46].
**Medulloblastoma**	S, V	*In vitro*—reversal of ABCB1-mediated resistance to etoposide by V [28]. S enhanced lethality of vincristine, etoposide and cisplatin [29].
**Melanoma**	S	*In vivo* reduction in MDSC numbers and inflammatory microenvironment in transgenic model [35].
**MM**	S, V	*In vitro* both drugs potentiated effect of EGCG on MM cell lines. *In vivo* V + EGCG improved survival in murine model [37].
**Pancreatic**	S	*In vivo*—reduction in MDSC numbers and increased immune response in PDAC model [36].
**Prostate**	S	*In vitro*, *in vivo*—combination with doxorubicin increased apoptosis, reduced tumour growth [17]. Slowed tumour growth in a TRAMP-C1 model [22].
**RMS**	S	*In vitro*, *in vivo*—combination with CNP potentiates anti-proliferative effects in cell lines and patient samples. Slowed tumour growth in xenograft model [51].
**Thyroid**	S, T	*In vitro*—both drugs reduce cell viability and migration in patient samples [49].

For drug S = sildenafil, T = tadalafil and V = vardenafil.

**Table 4.** Clinical trials in cancer using PDE5 inhibitors.

Identifier	Phase	Drug	Details
NCT02998736	I	T	The PERIOP-04 trial is a single arm, open-label trial of tadalafil and influenza virus in patients undergoing surgical resection of a primary abdominal malignancy. Tadalafil, at a dose of 20 mg/day, is administered for 16 days, starting 5 days before surgery and continuing to day 10 after surgery. A single dose of influenza vaccine (Agriflu) is administered on the day of surgery. While the primary outcomes are safety and toxicity related, secondary outcomes will assess post-surgical reduction in NK cell cytotoxicity.
NCT02466802	I	S	This is a dose finding study investigating the combination of sildenafil and regorafenib in patients with progressive advanced solid tumours
NCT01903083	I	T	A trial of tadalafil and chemoradiotherapy in borderline resectable and locally advanced pancreatic cancer. Patients are treated with tadalafil at 2.5 mg/day for 21 days in combination with three doses of IV gemcitabine. On study day 22, patients will receive the first of three planned doses of radiation therapy and continue daily tadalafil. Patients will then be evaluated to determine if they are candidates for pancreaticoduodenectomy. Patients who are not candidates will continue daily tadalafil and receive gemcitabine chemotherapy. Patients who have surgery will resume daily tadalafil and gemcitabine chemotherapy following recovery from surgery. The primary outcome is safety. Secondary outcomes are degree of immune infiltration in resected tissue and quantification of T cell numbers pre- and post-treatment.
NCT01342224	I	T	In this pancreatic cancer trial the intervention is the addition of tadalafil to a 4-week course of vaccination with telomerase vaccine (GV1001) and GM-CSF by injection, along with a cycle of gemcitabine chemotherapy (IV). This is followed by concurrent radiation therapy and low-dose intravenous (IV) gemcitabine chemotherapy given twice weekly followed by one additional dose of vaccine. Patients who are then assessed as resectable may undergo surgery. The primary outcome is safety, but secondary outcomes will assess immune infiltration in tumour tissue and tumour response.
NCT02279992	I	V	This is an early phase I trial to assess the combination of vardenafil and carboplatin in patients with recurrent primary gliomas or brain metastases. Patients will be randomly assigned to receive either 20 mg vardenafil, followed by carboplatin or carboplatin alone prior to tumour resection. Carboplatin levels will be determined from both serum and resected tumour tissue to assess whether vardenafil can increase the level of carboplatin that crosses the blood/brain barrier.
NCT03238365	I	T	In this two-arm phase I window of opportunity trial patients with recurrent HNSCC are treated with either the anti-PD1 checkpoint inhibitor nivolumab or nivolumab combined with daily tadalafil. The primary outcome is pre- and post-surgical immune cell polarisation (Th1/Th2 and M1/M2). A secondary outcome is the degree of immune cell infiltration in resected tumour tissue.
NCT03259516	I/II	S	This is a phase I/II multi-arm trial assessing the combination of nivolumab and a range of drugs in patients with a myelodysplastic syndrome. One of the arms of the trial will assess the combination of nivolumab, cytarabine and sildenafil (20 mg TID).
NCT02544880	I/II	T	This is also a phase I/II trial of the combination of anti-tumour vaccines and tadalafil in resectable and/or recurrent HNSCC. Phase I is a single arm study assessing safety and tolerability of tadalafil with anti-MUC1 and anti-influenza vaccines. Phase II is a placebo controlled multi-arm study looking at anti-MUC1 and anti-influenza vaccines with and without perioperative tadalafil and placebo vaccine with tadalafil. The tadalafil dose is 10, 15 or 20 mg/day, depending on patient bodyweight. For the phase II study the primary outcome is tumour-specific immune response, with recurrence-free survival as the secondary outcome.
NCT01817751	II	S	An open label trial of sildenafil, sorafenib and valproic acid in patients with recurrent high-grade glioma. Patients are treated with this all-oral combination of drugs on a 4-week cycle, with sildenafil prescribed twice daily (dose unspecified). The primary outcome is the 6-month PFS rate, with sub-group analysis based on PDGFRa expression. Secondary outcomes include overall survival.
NCT01858558	II	T	This is a randomised open-label two arm study in high-risk MM. Patients in both arms of the trial are treated with anti-pneumonia vaccine (PrevNar 13), autologous stem cell transplant, tadalafil and 60-days post-transplant lenalidomide. Additionally, patients in the treatment arm will also be administered activated marrow-infiltrating lymphocytes. The primary outcome is 2-year PFS, with OS as one of the secondary outcomes.
NCT02335242	II	S	A randomised, blinded, placebo-controlled trial of daily sildenafil (for 20 weeks) in infants and children (6 months–10-years) with lymphatic malformations in the head and neck region. Primary outcome is change in lesion volume; secondary outcome is change in lesion appearance.

Drug S = sildenafil, T = tadalafil and V = vardenafil.

**Table 5.** Summary of evidence by cancer type.

Cancer type	*In vitro*	*In vivo*	Case report/Trial
**Breast**	[26, 27, 39, 93]	[31, 32, 34]	
**CLL**	[14]		[14]
**Colorectal**	[23, 25, 33]	[19, 21, 23, 25, 47]	NCT02998736
**EAC**		[52]	
**Gastric**	[18]		
**Glioma**	[33]	[26, 27]	NCT02279992 NCT01817751
**HNSCC**	[48]	[48]	[61, 62] NCT03238365 NCT02544880
**Liver (hepatocellular carcinoma)**	[33, 47]	[47]	
**Lung cancer**	[40, 42, 45]	[40, 42]	
**Lymphatic malformations**			NCT02335242
**Lymphoma**	[20]	[20, 21, 46]	
**Medulloblastoma**	[28, 29, 33]		
**Melanoma**		[35]	[64]
**MM**	[37]	[37]	[59] NCT01858558
**Myelodysplastic syndrome**			NCT03259516
**Pancreatic**		[36]	NCT01903083 NCT01342224
**Penile squamous cell carcinoma**			[58]
**Prostate**	[17]	[22, 17]	
**RMS**	[51]	[51]	
**Thyroid**	[49]		
**Waldenstrom’s macroglobulinemia**			[53, 54]
